# How Much Is the Dose Varying between Follow-Up CT-Examinations Performed on the Same Scanner with the Same Imaging Protocol?

**DOI:** 10.1371/journal.pone.0152961

**Published:** 2016-04-06

**Authors:** Saravanabavaan Suntharalingam, Franz Ferdinand Stecker, Nika Guberina, Adrian Ringelstein, Thomas Schlosser, Jens Matthias Theysohn, Michael Forsting, Kai Nassenstein

**Affiliations:** Department of Diagnostic and Interventional Radiology and Neuroradiology, University Hospital Essen, Essen, Germany; Mizoram University, INDIA

## Abstract

**Purpose:**

To investigate the dose variation between follow-up CT examinations, when a patient is examined several times on the same scanner with the identical scan protocol which comprised automated exposure control.

**Material and Methods:**

This retrospective study was approved by the local ethics committee. The volume computed tomography dose index (CTDI_vol_) and the dose-length-product (DLP) were recorded for 60 cancer patients (29 male, 31 female, mean age 60.1 years), who received 3 follow-up CT examinations each composed of a non-enhanced scan of the liver (LI-CT) and a contrast-enhanced scan of chest (CH-CT) and abdomen (AB-CT). Each examination was performed on the same scanner (Siemens Definition FLASH) equipped with automated exposure control (CARE Dose 4D and CARE KV) using the identical scan protocol.

**Results:**

The median percentage difference in DLP between follow-up examinations was 9.6% for CH-CT, 10.3% for LI-CT, and 10.1% for AB-CT; the median percentage difference in CTDI_vol_ 8.3% for CH-CT, 7.4% for LI-CT and 7.7% for AB-CT (p<0.0001 for all values). The maximum difference in DLP between follow-up examinations was 67.5% for CH-CT, 50.8% for LI-CT and 74.3% for AB-CT; the maximum difference in CTDI_vol_ 62.9% for CH-CT, 47.2% for LI-CT, and 49% for AB-CT.

**Conclusion:**

A significant variance in the radiation dose occurs between follow-up CT examinations when the same CT scanner and the identical imaging protocol are used in combination with automated exposure control.

## Introduction

The use of computed tomography (CT) has risen nearly 20-fold in the United States since the 1980s [1]. Medical imaging now accounts for more than 50% of radiation exposure in the United States, half of which is related to CT [2, 3].

To minimize the potential risks associated with radiation exposure, different techniques have been established to reduce the radiation dose of CT imaging [4]: While iterative image reconstruction allows significant dose reductions compared to filtered back projection while maintaining the image quality [5, 6], automated tube voltage selection (ATVS) and automated tube current modulation (ATCM), also known as automated exposure control, enable CT imaging at the lowest dose necessary to achieve a predefined image quality regardless of the patient size or the attenuation characteristics of the body part being scanned [7–10]. Moreover, adaptive dose shielding reduces unnecessary radiation exposure along the z-axis before and after the image volume by using a collimator, which closes asymmetrically at the beginning and the end of the examination [11, 12].

Despite of all these automated dose reduction techniques, wide variations in the radiation exposure have been reported for similar CT examinations amongst different departments [13–18]. For example, Dougeni et al. reported that the effective dose of similar CT examinations varied up to 32-fold between different sites [18]; Marin et al. reported recently a significant variation in the radiation dose of pediatric cervical -spine CT examinations of more than 2.5-fold between different hospitals [19]. These variations have been explained by different scanner types, by different scanning protocols (tube voltage, tube current, tube rotation speed, pitch, collimation, filtration, etc.) and by major differences in the patient’s positioning (e.g. arms up vs. arms down).

To the best of the authors’ knowledge, the question, if the radiation exposure is the same, when all these parameters are kept identical, has not been addressed so far. From a theoretical point of view, the same radiation exposure could be expected, when all imaging parameters including patient’s positing are absolutely the same. However, between follow-up examinations small differences in the patient’s positioning as well as in the positioning and length of the scan are unavoidable, so that the conditions of follow-up examinations are never hundred percent identic even when all general scan parameters are kept unchanged and the same CT scanner is used. Due to the fact, that recent studies showed, that already small differences in the patients’ centering have a major impact on the radiation dose when ATCM is used, significant differences in the radiation exposure must be suspected in clinical routine even between follow-up CT examinations on the same CT scanner, when automated exposure control is used. However, this has not been investigated so far, despite the fact that the knowledge of this variation and its causes may allow to reduce radiation exposure of CT in clinical routine.

Thus, the aim of the present study was to investigate the dose variation between follow-up CT examinations performed on the same scanner with a fixed scan protocol, which comprised automated exposure control, and to identify its causes.

## Material and Methods

This retrospective single center study was approved by the University Hospital Essen ethics committee. Written informed consent was waived by the Institutional Review Board due to the retrospective character of the study and anonymized data evaluation.

### Patients

Sixty patients (31 female, 29 male, mean age at time of first CT examination: 60.1 ± 13.4 years) suffering from cancer, who received three follow-up CT examinations each composed of a non-enhanced scan of the liver (LI-CT) and a contrast-enhanced scan of chest (CH-CT) and abdomen (AB-CT) on the same CT scanner with the same imaging protocol were retrospectively selected from our RIS/PACS database (Centricity RIS Version 5.0, Centricity PACS Version 4.0, GE Healthcare IT, Barrington, Illinois, USA). For patient selection a list of all patients, who received a CT examination in our department on our dual-source CT after March 2014, was generated and ordered by the date of examination. The first 60 consecutive patients, who fulfilled the above mentioned inclusion criteria, were selected and evaluated for the present study. The median time interval between consecutive scans was three months (range 4 to 16 weeks).

### Image acquisition

All examinations were performed on a second generation dual-source scanner (Somatom Definition Flash, Siemens Healthcare, Forchheim, Germany) equipped with ATCM (CARE Dose 4D) and ATVS (CARE kV), and all patients were examined in supine position with both arms up. For planning of the scans a scout view was acquired in anterior-posterior direction during inspiratory breath-hold that covered the patient from the neck to the thighs (tube voltage 120 kV, tube current 35 mA). For non-contrast-enhanced imaging of the liver, the upper abdomen was imaged from the diaphragm to the lower margin of the liver. For ATVS a quality reference tube voltage of 120 kV was used. For ATCM a quality reference tube current-time product of 170 mAs was used and the algorithm was set to the mode “dose saving optimized for non-contrast-enhanced imaging” (number 3 on the 12-point scale of CARE kV, which is used to indicate the type of exam being performed). For contrast-enhanced imaging 100 ml Iobitridol with 350 mg iodine per ml (Xenetix 350, Guerbet, Roissy CdG Cedex, France) was injected in an antecubital vein via a 18G venous cannula with a flow rate of 3 ml/sec followed by a chaser bolus of 30 ml physiologic salt solution with a flow rate of 3 ml/sec using an automated contrast injector (Medrad Stellant, Medrad Inc., Warrendale, Pennsylvania, USA). Imaging of the chest was started 30 seconds after contrast injection. For ATVS and ATCM a quality reference tube voltage of 120 kV and a quality reference tube current-time product of 100 mAs were used, and ATVS was set to the mode “dose saving optimized for contrast-enhanced imaging” (number 7 on the 12-point scale of CARE kV). Seventy seconds after contrast injection the entire abdomen was imaged. A quality reference tube voltage of 120 kV and a quality reference tube current-time product of 170 mAs were used, and ATCM was set to the mode “dose saving optimized for contrast-enhanced imaging” (number 7 on the 12-point scale of CARE kV). All scans were acquired during inspiratory breath-hold with 128 x 0.6 mm detector configuration, 38.4 mm beam collimation, a pitch of 0.6, and a tube rotation time of 0.5 sec. For image reconstruction Sinogram Affirmed Iterative Reconstruction (SAFIRE, Siemens Healthcare, Forchheim, Germany) was used with a moderate strength of 2 (available strength of SAFIRE: 1 to 5, whereat a higher number implies a stronger noise reduction).

### Phantom measurements

A commercially available anthropomorphic sectional phantom of the male torso (Radiology Support Devices Inc., Long Beach, CA, USA) was examined on the same dual-source scanner that was used for patients’ examinations using the same scan protocol that was used for contrast-enhanced imaging of the patients’ chest and abdomen. First of all, chest and abdomen CT scans were acquired four times in which all scan parameters remained absolutely the same. In a second series of measurements only the table height was varied (10.4 cm, 12.4 cm, 14.4 cm, 16.4 cm, 18.4 cm). In a last series of phantom measurements the position of the phantom on the table was varied (eccentric positioning of the phantom on the table, lifting one side of the phantom by approximately 20° and by 90°) while the table height was set to the height of the initial phantom measurements (14.4 cm).

### Assessment of radiation dose

The volume CT dose index (CTDI_vol_), which was automatically calculated for a 32 cm body phantom by the CT scanner, and the dose-length-product (DLP) was recorded for each scan by using the dose monitoring program Radimetrics Enterprise Platform Ver. 2.3 (Bayer HealthCare, Indianola, Pennsylvania, USA).

### Registration of influencing parameters of radiation exposure

**Fig 1:**
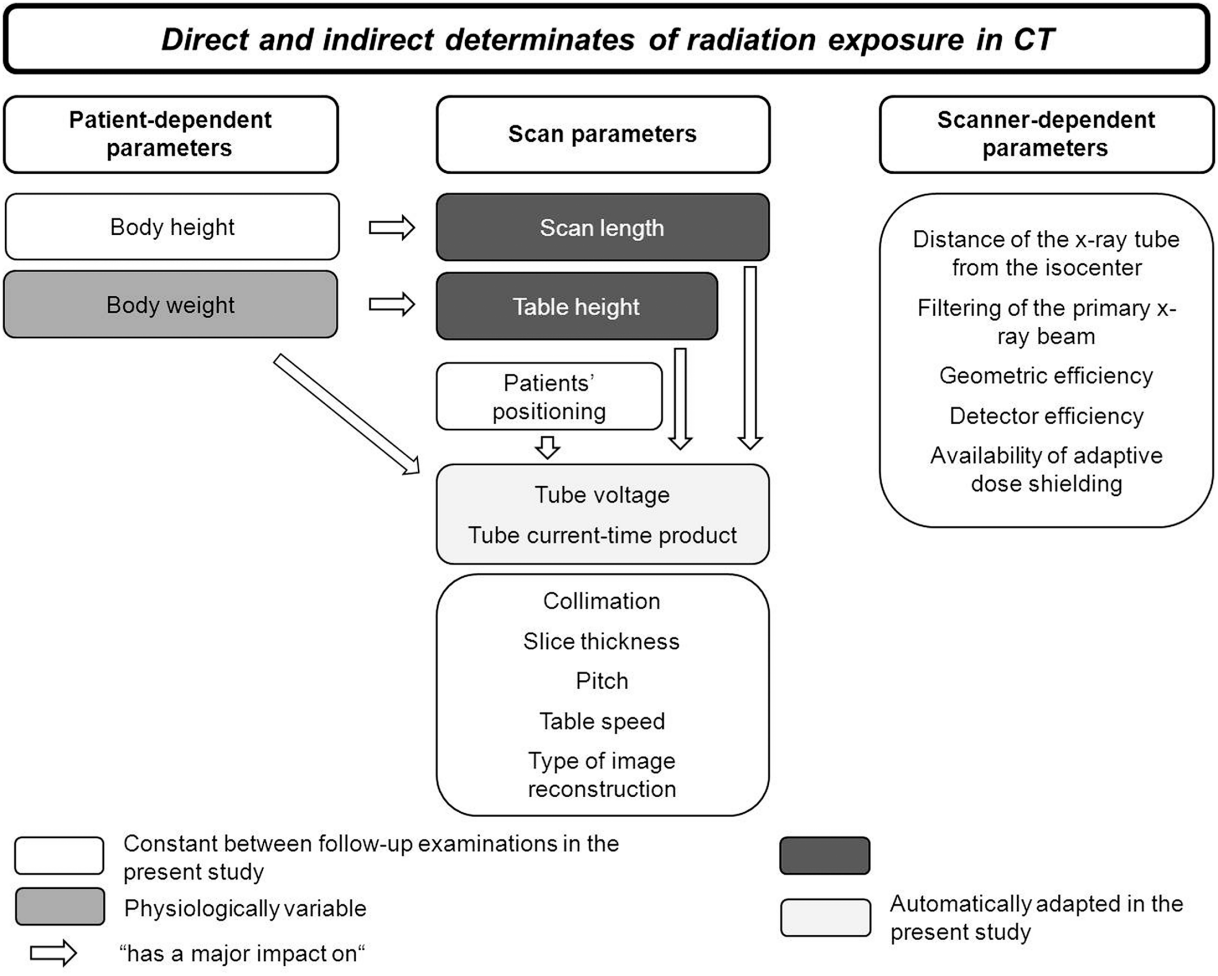
Schematic summary of the direct and indirect determinates of the radiation exposure in CT.

Due to the design of the present study, only three parameters varied independently between the follow-up examinations—the scan length, the table height, and the patient’s weight—while all other parameters were kept constant or were automatically adjusted by the scanner (tube voltage, tube current-time product) (Fig 1). The independent variables scan length and the table height were recorded, and the maximal effective diameter of the abdomen was calculated as surrogate parameter for patients’ weight [20, 21] in accordance to the definition in AAPM report 204 using Radimetrics [22] for each examination. Moreover, the tube voltage as well as the mean tube current-time product (mean mAs), which had been adjusted automatically by ATVS and ATCM in dependency of scan length, table height, and patient’s weight, was recorded for each scan.

### Statistical analysis

Statistical analysis was performed using MedCalc Version 13.3 (MedCalc Software, Mariakerke, Belgium). The differences between CTDI_vol_ and DLP at the different examination times were calculated, as well as the differences between the above mentioned influencing factors. Since all patients received three follow-up CT examinations, three difference values were generated for each parameter (e.g. DLP _second exam_−DLP _first exam_, DLP _third exam_−DLP _second exam_, DLP _third exam_−DLP _first exam_). For statistical analysis of the differences between the follow-up examinations, the absolute values of the above mentioned parameters were used, while for statistical analysis of causality between changes in radiation exposure and influencing factors the algebraic sign was considered. To test for normal distribution a D’Agostino-Pearson test was used. Normal distributed date are given as mean ± standard deviation (SD), non-normally distributed as median and interquartile range (IQR). Categorical data are expressed as absolute numbers. A signed-rank sum test for one sample was used to test the hypothesis, that the differences in DLP and CTDI_vol_ between follow-up examinations were different from 0. For correlation analysis Pearson correlation coefficients (r) were calculated in case of normal distributed data, and Spearman rang correlation coefficients (ρ) in case of non-normally distributed data. 95% confidence intervals (CI) were calculated for means, medians and correlation coefficients. A correlation with a correlation coefficient between 0.1 and 0.3 was rated as weak, with a coefficient between 0.4 and 0.6 as moderate, with a coefficient between 0.7 and 0.9 as strong, and with a coefficient of 1.0 as perfect. Stepwise multiple linear regression analysis, which attempts to model the relationship between two or more explanatory variables and a response variable by fitting a linear equation to observed data [23] was performed for analysis of the relationships between the influencing parameters of radiation exposure and the dose parameters (DLP, CTDI_vol_). The coefficient of determination (R^2^), which is the proportion of the variance in the response variable that is predictable from the explanatory variables [23], was calculated for each multiple linear regression analysis.

## Results

### Radiation exposure in patients and its variation between follow-up examinations

The median CTDI_vol_ was 5.9 mGy for chest CT (CH-CT), 8.1 mGy for non-enhanced CT of the liver (LI-CT), and 7.7 mGy for abdominal CT (AB-CT); the median DLP was 226 mGy*cm for CH-CT, 183 mGy*cm for LI-CT, and 363.5 mGy*cm AB-CT (for details see Table 1).

**Table 1:** Radiation dose (CTDI_vol_, DLP), manually (scan length, table height) or automatically (tube current, mean mAs) adjusted scan parameters as well as the maximal effective abdominal diameter as surrogate for patient’s weight of the 3 CT follow-up examinations each composed of a non-contrast-enhanced scan of the liver and a contrast-enhanced scan of the chest and abdomen in 60 patients.

	Contrast-enhanced CT of the chest	Non-enhanced CT of the liver	Contrast-enhanced CT of the abdomen
Number of examinations (n)	180	180	180
CTDI_vol_ [mGy]			
Median:	5.9	8.1	7.7
95% CI for the median:	5.4 to 6.4	7.6 to 8.9	7.1 to 8.2
IQR:	2.4	4.3	3.1
DLP [mGy*cm]			
Median:	226.0	183.0	363.5
95% CI for the median:	207.2 to 241.0	173.4 to 197.6	329.7 to 394.0
IQR:	130.0	108.5	180.5
Scan length [cm]			
Mean:	39.1	22.8	48.2
95% CI for the mean:	38.3 to 39.9	22.3 to 23.2	47.6 to 48.7
SD:	5.5	3.0	3.8
Table height [cm]			
Mean:	16.2	16.2	16.2
95% CI for the mean:	15.9 to 16.5	15.9 to 16.5	15.9 to 16.5
SD:	1.9	1.9	1.9
Tube voltage			
80 kV (n)	1	0	0
100 kV (n)	168	38	179
120 kV (n)	11	111	1
140 kV (n)	0	31	0
Mean mAs			
Median:	80.5	81.5	114.3
95% CI for the median:	75.8 to 86.7	78.6 to 83.5	105.8 to 119.9
IQR:	35.7	23.3	45.9
Max. effective diameter [cm]			
Mean:	32.2	32.2	32.2
95% CI for the mean:	31.6 to 32.7	31.6 to 32.7	31.6 to 32.7
SD:	3.6	3.6	3.6

Abbreviations: CTDI_vol_ = volume computed tomography dose index, DLP = dose-length-product, CI = confidence interval, IQR = interquartile range, SD = standard deviation.

The median difference in the CTDI_vol_ between follow-up examinations was 0.4 mGy for CH-CT, 0.65 mGy for LI-CT, and 0.59 mGy for AB-CT; the median difference in DLP was 21 mGy*cm for CH-CT, 19 mGy*cm for LI-CT, and 37 mGy*cm for AB-CT (for details including the range of the values see Table 2), whereas statistical analysis showed a significant difference (p < 0.0001) from 0 for all values.

**Table 2:** Differences between the follow-up examinations in radiation exposure, scan parameters and maximal effective abdominal diameter.

	Contrast-enhanced CT of the chest	Non-enhanced CT of the liver	Contrast-enhanced CT of the abdomen
Δ CTDI_vol_ [mGy]			
Median:	0.4	0.65	0.59
95% CI for the median:	0.37 to 0.50	0.51 to 0.73	0.51 to 0.71
IQR:	0.63	0.78	0.88
range:	0 to 5.5	0 to 5.8	0 to 5.0
Δ DLP [mGy*cm]			
Median:	21.0	19.0	37.0
95% CI for the median:	17.4 to 23.6	15.4 to 21.0	27.4 to 42.0
IQR:	31.0	23	50.5
range:	0 to 194	0 to 174	0 to 315
Δ scan length [cm]			
Median:	1.4	1.3	1.5
95% CI for the median:	1.1 to 1.6	1.0 to 1.5	1.2 to 2.0
IQR:	1.7	1.7	2.8
Δ table height [cm]			
Mean:	1.1	1.1	1.1
95% CI for the median:	0.9 to 1.3	0.9 to 1.3	0.9 to 1.3
IQR:	1.3	1.3	1.3
Δ tube voltage			
0 kV (n)	160	146	180
20 kV (n)	20	34	0
Δ mean mAs			
Median:	6.4	7.9	8.8
95% CI for the median:	5.4 to 7.8	6.1 to 9.1	7.1 to 10.4
IQR:	16.9	12.8	12.8
Δ max. effective diameter [cm]			
Median:	0.5	0.5	0.5
95% CI for the median:	0.4 to 0.6	0.4 to 0.6	0.4 to 0.6
IQR:	0.7	0.7	0.7

Abbreviations: CTDI_vol_ = volume computed tomography dose index, DLP = dose-length-product, CI = confidence interval, IQR = interquartile range.

**Fig 2:**
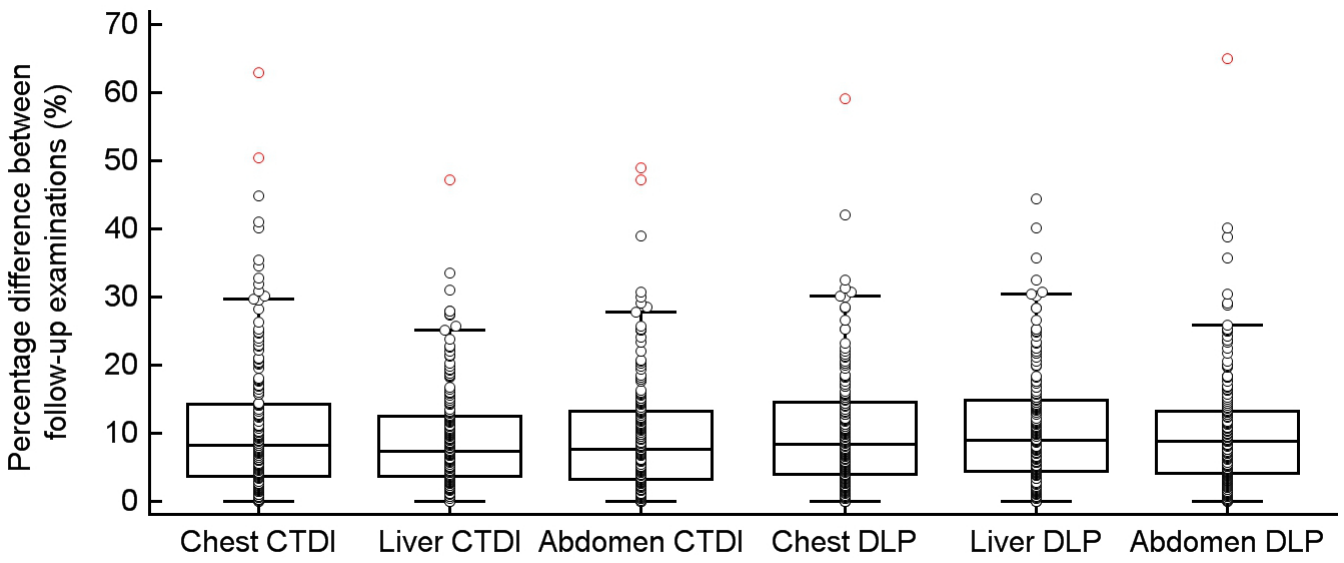
Box- and Whisker-plots of the percentage differences in CTDI_vol_ and DLP between follow-up examinations.

The median percentage difference in the CTDI_vol_ between follow-up examinations (Fig 2) was 8.3% for CH-CT (IQR: 10.6%, range: 0–62.9%), 7.4% for LI-CT (IQR: 8.8%; range: 0–47.2%), and 7.7% for AB-CT (IQR: 9.9%, range: 0–49%); the median percentage difference in DLP between follow-up examinations (Fig 2) was 9.6% for CH-CT (IQR: 10.4%, range: 0–7.3%), 10.3% for LI-CT (IQR 12.2%, range: 0–67.5%), and 10.1% for AB-CT (IQR: 10.4%, range: 0–74.3%), whereas statistical analysis showed a significant difference (p < 0.0001) from 0 for all values.

### Relation between differences in radiation exposure and differences in imaging parameters

A detailed correlation analysis between the differences in the radiation dose and all imaging parameters, which varied between the follow up examination are given in Table 3.

**Table 3:** Correlation analysis between the differences in the radiation exposure and the differences in the parameters that varied between the follow up examinations.

correlation between	Contrast-enhanced CT of the chest	Non-enhanced CT of the liver	Contrast-enhanced CT of the abdomen
Δ CTDI_vol_ and Δ table height			
ρ	-0.5	-0.63	-0.68
95% CI of ρ	-0.6 to -0.38	-0.71 to -0.53	-0.75 to -0.59
p	<0.0001	<0.0001	<0.0001
Δ CTDI_vol_ and Δ tube voltage			
ρ	0.52	0.42	0.172
95% CI of r	0.41 to 0.62	0.29 to 0.53	0.027 to 0.31
p	<0.0001	<0.0001	0.02
Δ CTDI_vol_ and Δ mean mAs*			
ρ	0.93	0.96	0.93
95% CI of r	0.91 to 0.95	0.95 to 0.97	0.91 to 0.95
p	<0.0001	<0.0001	<0.0001
Δ CTDI_vol_ and Δ max. effective diameter			
ρ	0.43	0.41	0.39
95% CI of ρ	0.31 to 0.55	0.28 to 0.53	0.26 to 0.51
p	<0.0001	<0.0001	<0.0001
Δ DLP and Δ scan length			
ρ	0.26	0.55	0.41
95% CI of ρ	0.12 to 0.39	0.44 to 0.65	0.28 to 0.52
p	0.0005	<0.0001	<0.0001
Δ DLP and Δ table height			
ρ	-0.45	-0.49	-0.62
95% CI of ρ	-0.56 to -0.33	-0.6 to -0.37	-0.7 to -0.52
p	<0.0001	<0.0001	<0.0001
Δ DLP and Δ tube voltage			
ρ	0.51	0.37	0.03
95% CI of r	0.39 to 0.61	0.24 to 0.49	0.02 to 0.31
p	<0.0001	<0.0001	0.03
Δ DLP and Δ mean mAs*			
ρ	0.82	0.67	0.87
95% CI of r	0.77 to 0.87	0.56 to 0.75	0.83 to 0.90
p	<0.0001	<0.0001	<0.0001
Δ DLP and Δ max. effective diameter			
ρ	0.3	0.39	0.43
95% CI of ρ	0.16 to 0.42	0.26 to 0.51	0.3 to 0.54
p	0.0001	<0.0001	<0.0001

Abbreviations: CTDI_vol_ = volume computed tomography dose index, DLP = dose-length-product, CI = confidence interval.

Note:

* Calculated only for cases in which the tube voltage was kept constant between follow up examinations.

Due to the design of the present study (Fig 1), only three parameters varied independently between the follow-up examinations: the scan length, the table height, and the patient’s effective abdominal diameter.

A weak positive correlation was found between differences in scan length and the differences in DLP between follow-up examinations for CH-CT, while a moderate positive correlation was found for LI-CT, and AB-CT (Table 3). Overall a moderate negative correlation was observed between differences in the table height and differences in CTDI_vol_, and DLP, respectively (Table 3). A weak to moderate positive correlation was observed between the differences in the patients’ effective abdominal diameter and the differences in CTDI_vol_, and DLP, respectively (Table 3).

**Fig 3:**
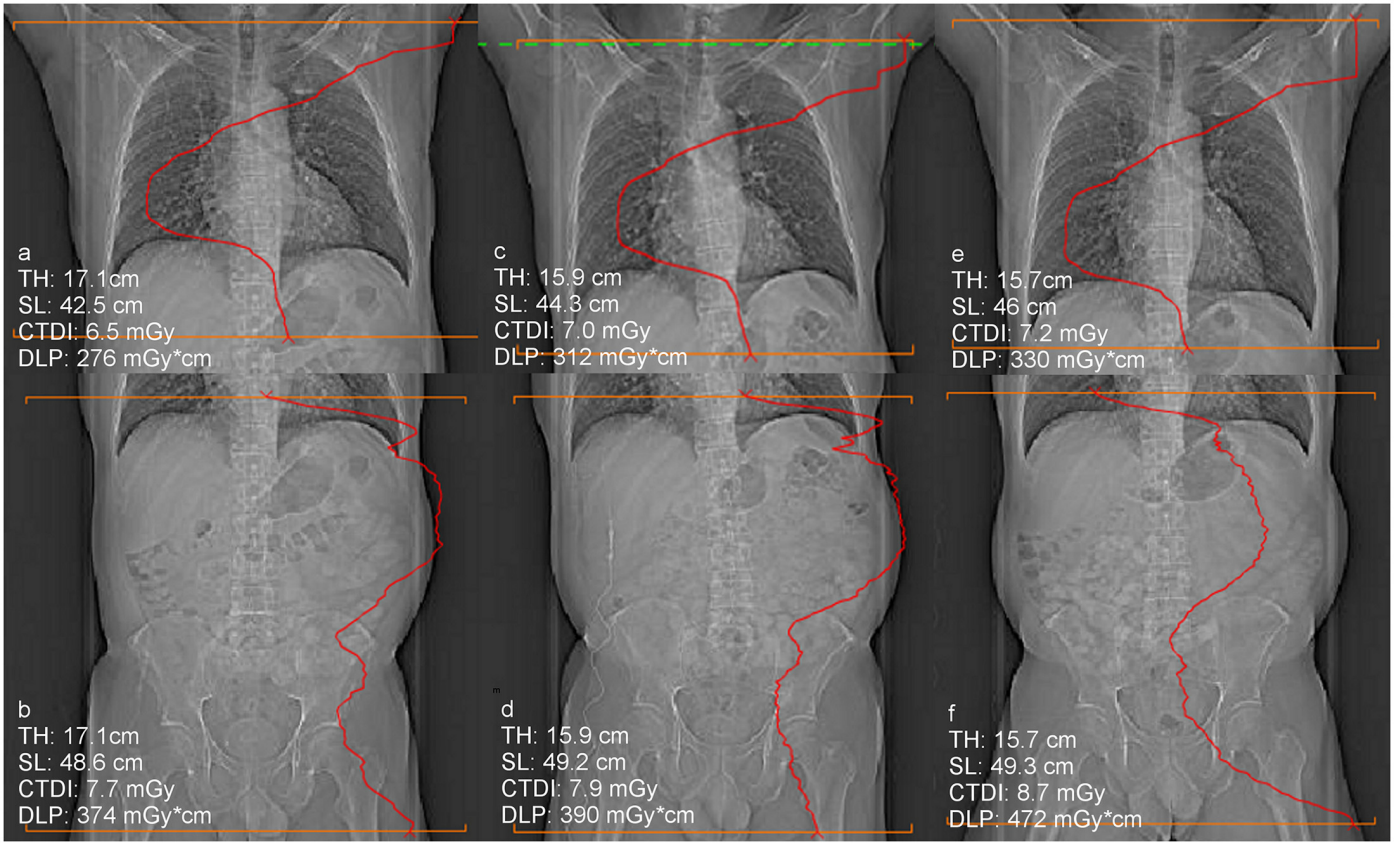
a-f: Scout views of three follow-up CT examinations of a 54 year old male patient with superimposed modulation of the tube current. While the differences in the radiation dose between the chest CT examinations can be explained by differences in the table height (TH) and the scan length (SL), the difference in dose between the abdominal CT scans d and f, cannot be explained by these factors due to the fact that in d and f the table height as well as the scan length had been virtually the same. However, the positioning of the thighs was different between these examinations, which resulted in a different tube current modulation and consecutively in different radiation dose.

Multiple regression analysis showed that changes in the table height and changes in the patients’ effective abdominal diameter determine the differences in CTDI_vol_ between follow-up examinations to 33% in CH-CT, to 62% in LI-CT, and to 66% in AB-CT (Table 4, Fig 3). Variations in table height, scan length and patients’ effective abdominal diameter determine the differences in DLP between follow-up examinations to 35% for CH-CT, to 67% in LI-CT, and to 68% in AB-CT (Table 4).

**Table 4:** Multiple linear regression analysis to predict differences in the radiation exposure between follow-up examinations.

		Contrast-enhanced CT of the chest		Non-enhanced CT of the liver		Contrast-enhanced CT of the abdomen	
		CTDI_vol_	DLP	CTDI_vol_	DLP	CTDI_vol_	DLP
Predictors							
	Δ scan length [cm]						
	coefficient	n.a.*	6.6	n.a.*	8.4	n.a.*	7.5
	p	n.a.*	<0.0001	n.a.*	<0.0001	n.a.*	<0.0001
	Δ table height [cm]						
	coefficient	-0.32	-12.4	-0.43	-9.0	-0.43	-20.7
p	<0.0001	<0.0001	<0.0001	<0.0001	<0.0001	<0.0001
Δ abdominal diameter [cm]						
coefficient	0.49	19.0	0.63	14.7	0.61	31.7
p	<0.0001	<0.0001	<0.0001	<0.0001	<0.0001	<0.0001
Multiple correlation coefficient		0.57	0.6	0.79	0.82	0.81	0.83
Coefficient of determination R^2^		0.33	0.35	0.62	0.67	0.66	0.68

Abbreviations: CTDI_vol_ = volume computed tomography dose index, DLP = dose-length-product, n.a. = not applicable.

Note:

* Not applicable due to the fact that CTDI_vol_ is per definition independent from the scan length.

### Phantom measurements

Repeated imaging with absolute consistency of all imaging parameters resulted into almost perfect constancy of CTDI_vol_ (CH-CT: 4.9 mGy for all measurements; AB-CT: 5.3 mGy for all measurements), and DLP (CH-CT: 178 mGy*cm in 2, and 179 mGy*cm in 2 measurements; AB-CT: 275 mGy*cm in 3, and 277 mGy*cm in 1 measurement). A perfect negative linear correlation was observed between changes in table height and changes in CTDI (CH-CT: r = -0.992; AB-CT: r = -0.998), and DLP (CH-CT: r = -0.991; AB-CT: r = -0.997). Changes in the phantom’s positioning while maintaining all other imaging parameters constant resulted in major changes in radiation exposure (Table 5).

**Table 5:** Influence of the phantom’s positioning on the CT table on the radiation dose. Except of the phantom’s positioning all other imaging parameters were kept constant between the measurements.

	Chest CT		Abdominal CT	
	CTDI_vol_	DLP	CTDI_vol_	DLP
baseline phantom examination	4.87 mGy	178 mGy*cm	5.29 mGy	275 mGy*cm
eccentric positioning of the phantom on the table	3.38 mGy	124 mGy*cm	4.26 mGy	222 mGy*cm
lifting one side of the phantom by approximately 20°	3.37 mGy	123 mGy*cm	4.26 mGy	222 mGy*cm
lifting one side of the phantom by approximately 90°	3.60 mGy	132 mGy*cm	4.22 mGy	220 mGy*cm

Abbreviations: CTDI_vol_ = volume computed tomography dose index, DLP = dose-length-product.

## Discussion

Several parameters have a direct or indirect effect on the radiation dose and image quality of CT, notably the beam energy (determined by the tube voltage), the photon fluence (determined by the tube current and the tube rotation time), the collimation, the pitch, the table speed as well as the used type of image reconstruction (filtered back projection vs. iterative image reconstruction) [24, 25]. Beside these parameters, device specific factors such as the distance of the x-ray tube from the isocenter, the detector efficiency, filtering of the x-ray beam, availability of adaptive dose shielding, as well as patient specific parameters notably body weight and height have an impact on the radiation dose and image quality of CT examinations [24, 25]. Due to the fact, that the effects of these parameters on radiation exposure, as well as on image quality are interlinked and complex [25, 26], it is not astonishing, that previous studies observed huge differences in the radiation exposure of “similar” CT examinations performed at different institutions (1–6).

Contrary to these inter-institutional studies, in which virtually all relevant parameters differed between the compared “similar” CT scans, all parameters were identic between the follow-up scans in the present study, except of the scan length, the table height and the patients’ maximal effective abdominal diameter (as surrogate for the patients’ weight). Nevertheless, our study showed for the first time that considerable differences in the radiation exposure can occur between virtually identic follow-up examinations (Fig 3a–3f).

Automated tube voltage selection (ATVS), as well as automated tube current modulation (ATCM) had been used in the present study, whereby the reference quality specifications (quality reference tube voltage and quality reference tube current-time product) were kept constant between the follow-up examinations. ATCM and ATVS have been developed to reduce the radiation dose while maintaining the image quality on a predefined quality level: In principle, ATCM achieves a dose reduction by adjusting the X-ray tube output to the patient’s size and shape [7, 27]. Practically ATCM can either be realized on refined analysis of the scout image, on a feedback circuit with near real-time adjustment of the tube current based on the attenuation values of the preceding image, or by a combination of both methods [25] like Siemens CARE Dose 4D, which had been used in the present study. Due to the fact, that the tube voltage has a considerable influence on radiation dose ATVS has been introduced in clinical practice [10, 28, 29]. In principle, lowering of the tube voltage leads in contrast enhanced imaging to a better contrast, since the low energy X-rays are better absorbed by the iodine than by the surrounding tissue [30]. However, since lowering the tube voltage results in increasing noise, the tube current requires usually an up-regulation to maintain the predefined image quality. Siemens CARE kV, which has been used in the present study, therefore aims to select the optimal combination of tube voltage and tube current for each patient according to the patient’s scout view and study objective. Based on the information of the patient’s attenuation along the patient’s longitudinal axis from the scout image, the required tube current is calculated for different tube voltages to reach the user defined image quality, and, thereafter, the combination with the lowest CTDI is used to perform the scan.

Since CARE Dose 4D as well as CARE KV obtain information from the scout view, differences between the scout views of follow-up examinations may have contributed to the observed differences in the radiation dose between follow-up examinations. In principle the following factors can modify the patient’s scout view: Differences in the patient’s positioning, loss or gain of weight, differences in the table height, as well as differences in the method of scout view acquisition. Due to the facts, that we acquired the scout view in a standardized way, the latter factor can be excluded. While the effect of changes in the body weight on the scout view and consecutively on automated exposure control and radiation exposure is obvious, the relevance of the table height is not similarly obvious, and, therefore, the significant impact of the table height on the radiation exposure had been reported quite recently [15, 31, 32]. However, the relationship of table height and radiation dose is rather simple: Due to the fact that the table height determines the distance between patient and X-ray source as well as the distance between patient and detector, the table height determines the magnification factor of the scout view, what in turn influences automated exposure control. Consistently, our phantom measurements showed a perfect correlation between differences in the table height and differences in the radiation exposure.

Our statistically analysis showed that the differences in the patients’ maximal effective abdominal diameter as surrogate for the patient’s weight, in the scan length, and in the table height determine the differences in the radiation exposure between follow-up examinations only to 33% to 68%. Since our phantom measurements showed, that even small differences in the phantom’s positioning on the examination table can cause major differences in the radiation exposure, small differences in the patients’ positioning (e.g. eccentric positioning on the table, tilted positioning) are in all likelihood another cause for the observed differences in the radiation dose between the follow-up examinations in patients. Moreover, differences in the positioning of the scan along the patients’ longitudinal (z) axis between follow-up examinations may have also contributed to the observed differences in radiation dose. Especially in chest CT this factor may have played a major role due to the fact that in chest CT differences in the positioning of the scan along the z-axis result in a different extent of inclusion of the shoulder girdle into the scan, what again has an immediate influence on ATCM and consecutively on the radiation dose due to the higher diameter of the shoulder girdle compared to the thorax. This consideration is supported by our finding that the extent of variation in radiation dose between follow-up examinations that is statistically not explained by variations in scan length, in table height and in the maximal effective abdominal diameter was significant higher for chest CT compared to liver and abdominal CT. Unreliability of ATCM and ATVS as further cause for the observed differences in the radiation dose between follow-up examinations can be excluded due to the fact that our phantom measurements showed an excellent reproducibility of the radiation exposure, when the phantom was scanned repeatedly with absolute identic scan parameters.

Our paper has some limitations. First and foremost, we used in the present study only one CT scanner. Due to the fact, that the algorithms used for automated exposure control differ between different manufacturers our results cannot be transferred easily to other implementations. Another potential limitation is that we did not assessed tumor growth or shrinkage in our patients, since it could not be excluded with certainty, that changes in the tumor burden have a certain effect on the patients’ attenuation characteristics, and, therefore, on radiation dose in CT

## Conclusion

A significant variance in the radiation dose can occur between follow-up CT examinations when the same CT scanner and the same imaging protocol are used in combination with automated exposure control. Our study showed that changes in the table height, changes in the scan length, small differences in the patient’s positioning, and small differences in the positioning of the scan range along the patient’s longitudinal axis are causes for the observed differences in radiation dose between similar follow-up examinations. Therefore, these factors should be carefully considered in clinical routine.
